# Comparison of Corneal Epithelial and Stromal Thickness Distributions between Eyes with Keratoconus and Healthy Eyes with Corneal Astigmatism ≥2.0 D

**DOI:** 10.1371/journal.pone.0085994

**Published:** 2014-01-28

**Authors:** Wen Zhou, Aleksandar Stojanovic

**Affiliations:** 1 SynsLaser Kirurgi AS, Tromsø, Troms, Norway; 2 Eye Department, University Hospital North Norway, Tromsø, Troms, Norway; Bascom Palmer Eye Institute, University of Miami School of Medicine, United States of America

## Abstract

**Purpose:**

To identify corneal epithelial- and stromal-thickness distribution patterns in keratoconus using spectral-domain optical coherence tomography (SD-OCT).

**Patients and Methods:**

We analyzed SD-OCT findings in 20 confirmed cases of keratoconus (group 1) and in 20 healthy subjects with corneal astigmatism ≥2 D (group 2). Epithelial and stromal thicknesses were measured at 11 strategic locations along the steepest and flattest meridians, previously located by corneal topography. Vertical mirrored symmetry superimposition was used in the statistical analysis.

**Results:**

The mean maximum keratometry measurements in groups 1 and 2 were 47.9±2.9 D (range, 41.8–52.8) and 45.6±1.1 D (range, 42.3–47.5), respectively, with mean corneal cylinders of 3.3±2.2 D (range, 0.5–9.5) and 3.6±1.2 D (range, 2.0–6.4), respectively. The mean epithelial thickness along the steepest meridian in group 1 was the lowest (37.4±4.4 µm) at 1.2 mm inferotemporally and the highest (59.3±4.4 µm) at 1.4 mm supranasally from the corneal vertex. There was only a small deviation in thickness along the steepest meridian in group 2, as well as along the flattest meridians in both groups. The stromal thickness distribution in the two groups was similar to the epithelial, while the stromal thickness was generally lower in group 1 than in group 2.

**Conclusions:**

SD-OCT provides details about the distribution of corneal epithelial and stromal thicknesses. The epithelium and stroma in keratoconic eyes were thinner inferotemporally and thicker supranasally compared with control eyes. The distribution pattern was more distinct in epithelium than in stroma. This finding may help improve the early diagnosis of keratoconus.

**Trial Registration:**

ClinicalTrials.gov NCT02023619

## Introduction

Identification of the corneal epithelial inferotemporal thinning and supranasal thickening by use of SD-OCT may help in the early diagnosis of keratoconus.

The corneal epithelium is a moldable [1] and active corneal layer that maintains the optical quality of the eye by remodeling itself to compensate for any changes in the stromal surface shape, e.g., those induced by keratorefractive surgery [2], scarring after corneal injuries or keratitis, or in keratoconus [3]–[6]. Information about the thickness distribution of the corneal epithelium may help identify stromal surface irregularities, as in subclinical keratoconus, before they are detectable on corneal topography [3], [4]. Mapping of the corneal epithelium has been attempted using various technologies, including immersion techniques such as high frequency ultrasound biomicroscopy [6], very high frequency digital ultrasound [3], [7]–[9] and confocal microscopy [3], [4], [10]. Non-contact optical coherence tomography (OCT) [5], [6], [11]–[13] has also been used.

OCT has been developed for non-invasive cross-sectional imaging in biological systems by using low-coherence interferometry to produce a two-dimensional image of optical scattering from internal tissue microstructures in a way that is analogous to ultrasonic pulse-echo imaging [14]. Spectral domain (SD), a newer generation OCT, seems to be reliable and reproducible enough to measure corneal epithelial thickness with sufficient axial resolution [12], in contrast to the measurements with the previous generation instruments based on time domain (TD) OCT [11], [12].

The current study evaluated epithelial and stromal thickness distributions measured by SD-OCT in eyes with keratoconus and in healthy eyes with ≥2.0 D of astigmatism. Recently, studies utilizing the same OCT technology [5], [13] compared keratoconic corneas with corneas of healthy subjects with unspecified amounts of astigmatism. The current study used healthy corneas with ≥2D of astigmatism as the control group to eliminate the influence of possible variations due to the astigmatism itself. This study aimed to define epithelial and stromal thickness–distribution-based variations that may help in early keratoconus detection.

## Patients and Methods

The protocol for this trial and supporting CONSORT checklist are available as supporting information; see Checklist S1 and Protocol S1.

### Ethics Statement

This study was approved by the regional ethics committee (Registry name: REK - Regionale Komiteer for Medisinsk Forsknings Etikk - MH Bygget, Universitetet i Tromsø, Norway. Registry number: 2013/758). The participants provided a written informed consent before the examination. The consent form was approved by the ethics committee. This study adhered to the tenants of the declaration of Helsinki.

### Patients

The participants were recruited from previously diagnosed keratoconus patients referred to the Eye Department of the University Hospital North Norway in Tromsø, Norway for corneal collagen cross-linking (CXL)-treatment and from healthy subjects seeking preoperative evaluation for refractive surgery at SynsLaser Kirurgi AS Tromsø, Norway. Patients were examined by the RTVue100 (OptoVue Inc. Fremont, California, USA) 26000-Hz SD-OCT using an add-on lens (CAM-L) mode, by Precisio (iVIS Technologies, Taranto, Italy) Scheimpflug-based topo/tomography, and by OPD-scan II (Nidek CO, LTD, Aichi, Japan) Placido-based topography. We analyzed SD-OCT corneal scans of 20 consecutive eyes with keratoconus (nine eyes categorized as stage 1, 7 eyes stage 2, and 4 eyes stage 3 on the Krumeich scale) (group 1) and of 20 consecutive healthy eyes with corneal astigmatisms ≥2 D, used as controls (group 2).

### Methods

**Figure 1:**
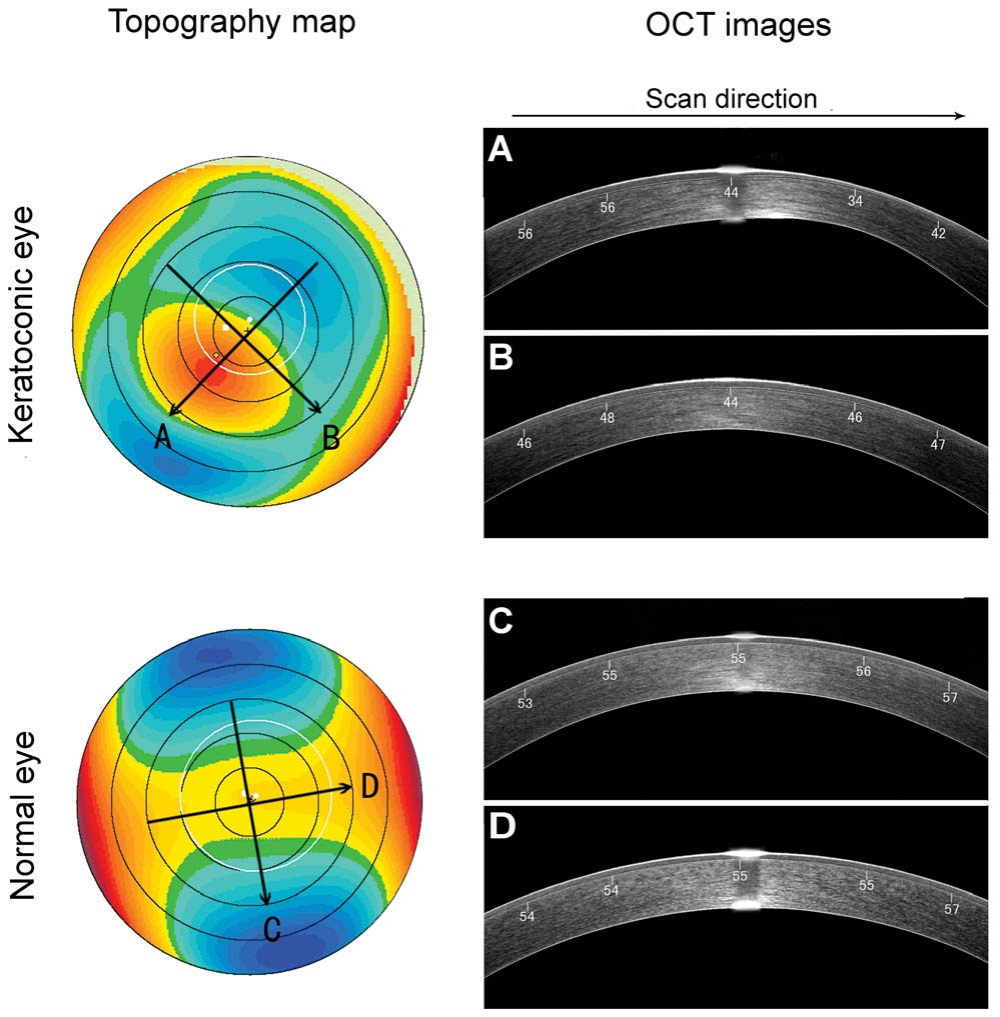
Corneal elevation topography and corneal optical coherence tomography (OCT) images. Anterior corneal elevation topography and cross-sectional corneal optical coherence tomography (OCT) images at the steepest (A, C) and flattest meridians (B, D) were taken for one keratoconic eye and one healthy eye with high corneal astigmatism.

The patients fixated on the target of the RTVue100 while 3 consecutive images were acquired to ensure measurement reproducibility. We analyzed two cross-sectional meridional corneal profiles obtained by SD-OCT along the steepest and the flattest meridians, previously located by Scheimpflug-topography. Figure 1 shows anterior corneal elevation topography and cross-sectional OCT images at both the steepest meridian (A, C) and the flattest meridian (B, D) for one keratoconic eye and one healthy eye with high corneal astigmatism.

To map the epithelial and stromal thicknesses, a software caliper tool from the RTVue100 was used. The following 11 points were measured in eyes from group 1: Seven points along the steepest meridian (corneal vertex, the points of the thinnest and thickest epithelia, as well as the points 1.5 mm and 2.5 mm from the corneal vertex) and 4 points along the flattest meridian (1.5 mm and 2.5 mm from the corneal vertex). Eleven corresponding points were measured in eyes from group 2. The amount of corneal astigmatism was measured from topography maps obtained by OPD-scan.

The sample size was decided by the power calculation founded on data from the previous studies analyzing corneal epithelial thickness in keratoconus and healthy eyes. For a significance level of 0.05 and a statistical power of 0.8, the equation for sample size is: N = 31.4×σ^2^/D^2^ [15], where σ is assumed standard distribution of each group and D is the minimum expected difference between two means. According to the preliminary data collected from comparable studies [5], [16], population σ and D are chosen to be 5.0 and 4.7 respectively, yielding a sample size N = 35.5. In the current study we have chosen a sample size 40.

Statistical analysis was performed using SPSS 13.0 software. The independent-samples t test was used to assess the difference in epithelial and stromal thickness at corresponding locations between the two groups and between different locations within the group. Descriptive statistics were carried out for all eyes using vertical mirrored symmetry superimposition: thickness values for left eyes were reflected in the vertical axis and superimposed onto the right eye values so that nasal/temporal characteristics could be combined.

## Results

We analyzed data from 20 keratoconic eyes of 15 subjects (10 men and 5 women) (group 1) and from 20 healthy astigmatic eyes of 15 subjects (7 women and 8 men) (group 2). The average ages were 28.5±6.5 years (range, 19–41 years) and 29.4±9.5 years (range, 15–46 years) for the two groups, respectively. The mean maximum keratometry measurements were 47.9±2.9 D (range, 41.8–52.8 D) and 45.6±1.1 D (range, 42.3–47.5 D), respectively. The average anterior corneal cylinders were 3.3±2.2 D (range, 0.5–9.5 D) and 3.6±1.2 D (range, 2.0–6.4 D) for group 1 and 2, respectively. The two groups were similar with respect to the amount of astigmatism (*P* = 0.54) and age (*P* = 0.74). The corneal and stromal thicknesses at each location are shown in Table 1.

**Table 1:** Epithelial and Stromal Thickness With Keratoconus and Controls (Mean±Standard Deviation).

		Epithelial (µm)		Stromal (µm)	
Distance from vertex (mm)		Keratoconus	Control	Keratoconus	Control
Steepest Meridian	−2.5	52.3±6.7	53.5±3.4	467.2±41.5	503.6±32.5
	−1.5	42.5±6.0	53.5±4.2	428.5±29.2	477.7±32.8
	−1.2	37.4±4.4	52.7±4.7	425.0±30.5	473.8±30.7
	+1.4	59.3±4.4	52.4±4.1	467.7±29.5	486.9±35.3
	+1.5	55.2±4.8	52.2±4.3	467.3±28.6	492.4±34.4
	+2.5	53.4±5.6	51.4±4.0	499.7±28.5	531.1±37.7
Corneal Vertex		45.7±3.8	52.6±4.2	428.5±31.4	470.1±31.2
Flattest Meridian	−2.5	53.2±3.9	51.2±4.1	467.0±30.6	491.7±32.0
	−1.5	49.0±4.6	51.2±3.9	437.9±25.5	473.5±32.1
	+1.5	51.1±4.1	53.0±4.8	464.8±35.7	490.8±37.2
	+2.5	53.2±3.6	52.1±3.4	500.0±30.1	520.1±41.8

For steepest meridian, “−” represents inferior to corneal vertex, “+” represents superior to corneal vertex. For flattest meridian, “−” represents temporal to corneal vertex, “+” represents nasal to corneal vertex.

### Epithelial thickness distribution

**Figure 2:**
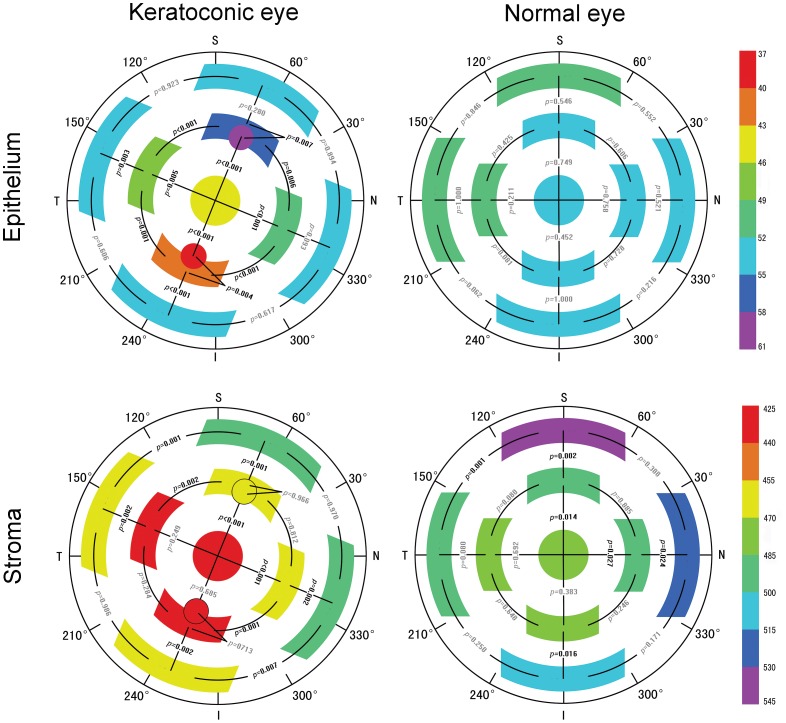
Topographic maps of the average epithelial and stromal thicknesses for keratoconic eyes and healthy eyes. The color scale represents the thickness in µm; I = inferior; N = nasal; S = superior; T = temporal.

The average angles of the steepest meridian were 67.8±14.7° (range, 50–105°) and 90.3±5.5° (range, 80–100°) for group 1 and group 2, respectively. The average epithelial thickness map in group 1 showed epithelial thinning inferotemporally and thickening supranasally (Figure 2). The thinnest and thickest epithelia were located 1.2 mm inferiorly and 1.4 mm superiorly from the corneal vertex, along the steepest meridian, respectively. The average epithelial thickness along the flattest meridian was increasing centrifugally, while it did not vary as much as along the steepest meridian. The average epithelial thickness map in group 2 showed small deviations in the distribution along the steepest meridian and the flattest meridian. Figure 2 (top) shows topographic maps of the average epithelial thicknesses for both groups.

### Comparison of epithelial thicknesses between the two groups

**Figure 3:**
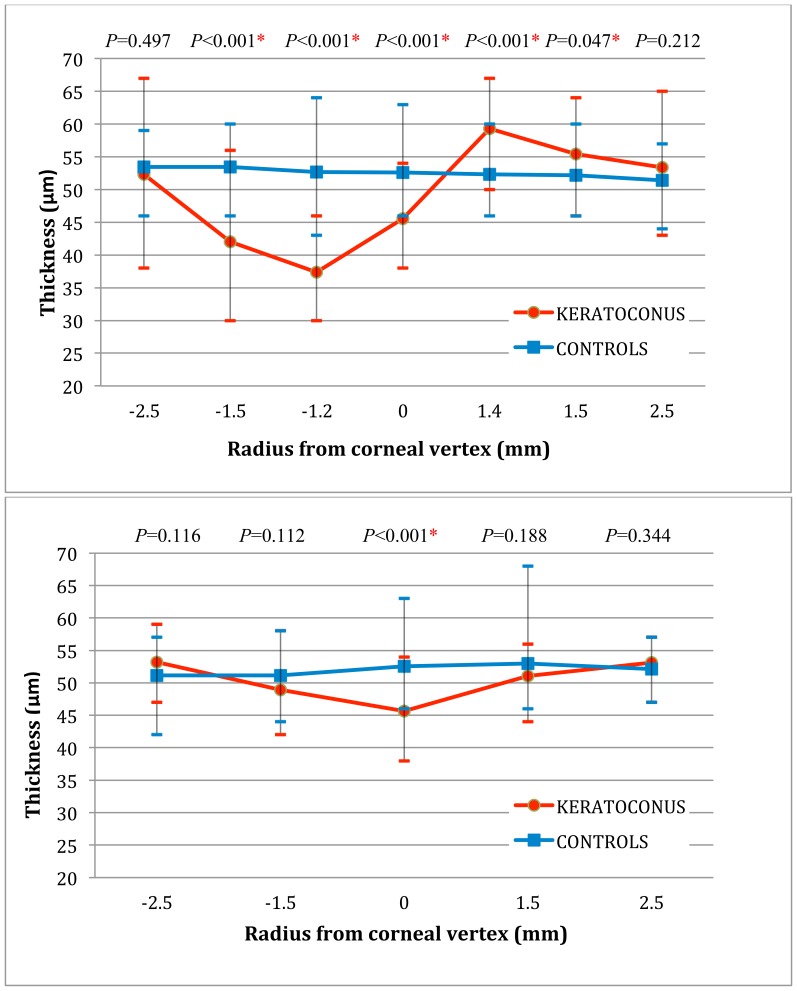
Comparison of average epithelial thicknesses for keratoconic and healthy astigmatic eyes. Top: Cross-sectional steepest-meridional average epithelial thicknesses for keratoconic and healthy astigmatic eyes. Bottom: Cross-sectional flattest-meridional average epithelial thicknesses for keratoconic and healthy astigmatic eyes.

On average, the epithelium in group 1 was thinner from the mid-inferior to centrum (p<0.001) and thicker mid-superiorly (p<0.001) along the steepest meridian when compared with group 2. There were no significant differences in the peripheral-superior and peripheral-inferior epithelial thicknesses between the two groups (*p* = 0.21 and 0.50, respectively). There was no significant difference between the two groups along the flattest meridian either, except for the location at the corneal vertex. The comparison of the cross-sectional steepest-meridional (top) and flattest-meridional (bottom) average epithelial thicknesses for the two groups is shown in Figure 3.

### Stromal thickness distribution

The maps of the average stromal thickness for both groups are shown in Figure 2 (bottom).

### Comparison of stromal thicknesses between the two groups

**Figure 4:**
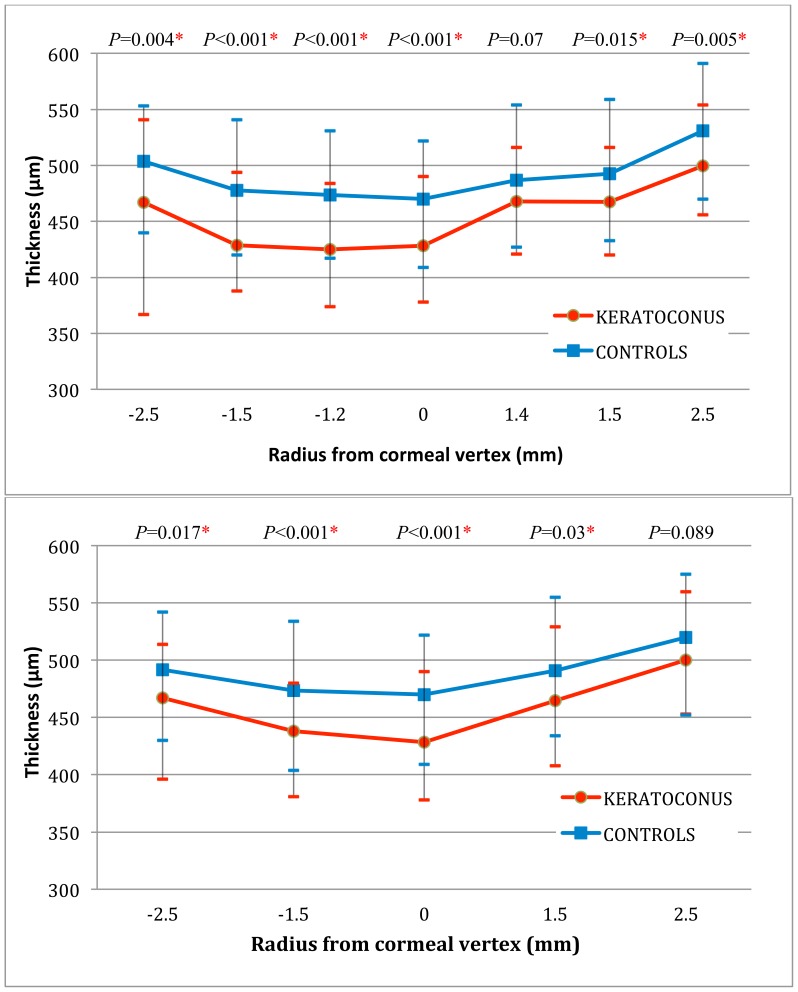
Comparison of average stromal thicknesses for keratoconic and healthy astigmatic eyes. Top: Cross-sectional steepest-meridional average stromal thicknesses for keratoconic and healthy astigmatic eyes. Bottom: Cross-sectional flattest-meridional average stromal thicknesses for keratoconic and healthy astigmatic eyes.

The comparison of the cross-sectional steepest-meridional (top) and flattest-meridional (bottom) average stromal thicknesses for the two groups is shown in Figure 4.

## Discussion

The phenomenon of epithelial remodeling has been described in eyes subjected to excimer laser ablations for correction of refractive errors, in eyes with keratoconus, and in connection with wearing overnight refractive therapy rigid contact lenses [5]–[7], [9], [10], [17], [18]. It has been theorized that eyelid friction may be chafing the corneal surface epithelium during blinking with the posterior surface of the semi-rigid tarsus providing a template for the outer shape of the epithelial surface [7], [8]. Hence, it seems that epithelial mapping may be applied to detect and diagnose keratoconus, since several variables based on epithelial thickness have shown good to excellent diagnostic power [13]. The epithelial pattern seems to depend on the severity of keratoconus [7] and it may also be used for monitoring the progress of the disease. Similarly, analysis of the epithelial thickness profile may be used to detect ongoing changes after keratoconus treatment with CXL and appears to be more sensitive than corneal topography [4], [6]. The epithelial thickness distribution has an impact on refraction [19] too, contributing to the corneal refractive power as well as to the power and axis of the astigmatism [14]. The cause of postoperative regression [19] after excimer laser treatments for correction of refractive errors may often be ascribed to compensatory epithelial remodeling, which also may be responsible for the lack of efficacy (less than 50%) of wavefront [5] or topography-guided [20] custom ablation profiles.

An epithelial thickness map may also provide key data for planning excimer laser treatments by providing information on the shape of the underlying stromal surface (by subtraction from the pachymetry map), which may be especially useful in transepithelial topography-guided surface ablation. Precise programming of the epithelial thickness into the ablation plan of transepithelial topography-guided treatments, instead of use of an anticipated value, will decrease the amount of stromal tissue removed by the ablation when the epithelium is thinner than anticipated, or it will guarantee that all of the planned stromal tissue is removed if the epithelium is thicker than anticipated [19], [21]. The data provided by the epithelial thickness mapping may also increase the safety and predictability of retreatments for myopic regression occurring after excimer laser surgery by providing information regarding whether the regression occurred due to the epithelial hyperplasia or corneal biomechanical changes [4], [8]. Using the same logic, data on the extent of central epithelial thinning, which commonly occurs with regression after hyperopic excimer laser surgery, may be crucial in determining whether a retreatment for hyperopic regression would be viable in a particular case.

The distribution of epithelial thickness has been studied previously using high frequency ultrasound biomicroscopy in normal corneas, untreated ectatic corneas, and in ectatic corneas previously treated with CXL [6]. Reinstein evaluated the Artemis very high-frequency (VHF) digital ultrasound system (ArcScan Inc., Morrison, Colo) extensively for measuring epithelial, stromal, and total corneal thickness distributions in healthy virgin eyes [8], in eyes that underwent laser refractive surgery [4], in keratoconus eyes [7], and to show the effectiveness of CXL [9]. Reinstein also introduced the term “epithelial doughnut pattern”, which is often used in the diagnosis of keratoconus [7]. In addition, according to Reinstein, epithelial thickness mapping may be an invaluable tool for detecting subclinical keratoconus in candidates for refractive surgery with normal anterior topography [3] as well as for excluding the diagnosis of KC in candidates with suspect topography [3]. The RTVue-100 SD-OCT has recently been used for epithelial thickness mapping in normal and keratoconus eyes [5], [13] as well as for comparing the epithelial thickness between normal non-lens wearers and rigid gas permeable (RGP) lens wearers and in RGP-wearing keratoconus patients [17]. VHF digital ultrasound features higher axial resolution and robust automatic mapping compared with SD-OCT technology. In addition, due to the immersion technique, the tear film component does not need to be dealt with; yet, it lacks control of fixation, the probe placement cannot be repeated, and it is difficult to ensure perpendicularity during the examination [16]. On the other hand, SD-OCT features a non-contact, quick and easy to perform examination that can be conveniently added to the existing examinations preceding corneal and refractive surgery [13].

Li [13] and Rocha [5] studied the epithelial thickness distribution in keratoconus using the same technology as the current study. However, they used healthy eyes with no regard to astigmatism and compared epithelial thickness between groups only; while, the current study used healthy eyes with ≥2 D corneal astigmatisms as the control group and epithelial thickness was compared both between groups and between locations within each group. In Li's study the epithelial thickness was automatically measured by computer software, while the current study employed visual inspection of the epithelial boundary and “manual” measurement of the epithelial thickness with the software caliper, eliminating the segmentation error with the automated epithelial boundary detection, which tends to occur in OCT mapping of keratoconus eyes [13]. In Rocha's study vertical and horizontal meridians were chosen [5], while the current study used the steepest and flattest meridians. Most of the cases in our group 1 had mild keratoconus and our control group consisted of eyes with a comparable amount of astigmatism to group 1 in order to achieve the highest possible sensitivity and specificity. Most of the other studies concerning epithelial thickness distribution in keratoconus [3], [5], [7], [22] did not categorize the stage of their keratoconus group and they used non-selected normal eyes as controls.

In the current study, the epithelial and stromal thicknesses were distributed similarly and the epithelium seemed to compensate for stromal anterior surface shape irregularities. In the keratoconus group, the stroma was elevated and stretched, showing thinning over the cone; while, the epithelium tended to have a smoother anterior topography and showed mid-peripheral thinning over the cone in addition to thickening superior to the cone. Epithelial thickness deviation was generally more pronounced and localized than stromal, showing its higher diagnostic sensitivity. The full ring of epithelial thickening surrounding the localized corneal thinning, as presented by Reinstein et al [3], [7], was not detected by Li and colleagues [13] or in the current study due to the low diameter of the studied area. However, since the keratoconus apex is located inside the central 5 mm of the cornea in the vast majority of keratoconic eyes [13], it seems that limiting the diameter of the epithelial mapping to only 5 mm may still be sufficient for keratoconus screening, but less so for pellucid marginal degeneration [13].

A point that potentially limits the quality of the data from the current study is the lack of quantification of the magnitude of the eyelid opening and the length of the corneal exposure during the examination. This may have led to some variability between subjects in the evaporation and movement of the tear film and could have induced some artifacts in the final epithelium thickness measurements [23]. Finally, the patients' contact lens history was not collected and the time between contact lens removal and examination was not recorded. However, as Haque stated, the epithelial thinning in keratoconus may only be slightly influenced by contact lens wear [17].

## Conclusions

It seems that fairly detailed epithelial and stromal thickness distribution maps can be compiled by the use of SD-OCT. The current study demonstrated that both epithelial and stromal thickness distributions in keratoconic eyes showed thinning along the steepest inferior hemimeridian, with the pattern being more distinct in the epithelium. Since the SD-OCT is a non-contact procedure that is easy to perform and is not uncomfortable for the patients, it has the potential to become part of the routine examination for the diagnosis of early keratoconus and for determining candidacy for refractive surgery.

## Supporting Information

Protocol S1 **Trial protocol.**
(PDF)

Checklist S1 **CONSORT Checklist.**
(DOC)

## References

[1] Lian Y, Shen M, Jiang J, Mao X, Lu P, et al (2013) Vertical and horizontal thickness profiles of the corneal epithelium and Bowman's layer after orthokeratology. Invest Ophthalmol Vis Sci 54: 691–696. 23221070 10.1167/iovs.12-10263

[2] Schmoll T, Unterhuber A, Kolbitsch C (2012) Precise thickness measurements of Bowman's layer, epithelium, and tear film. Optometry and vision science 89: E795–802. 22488267 10.1097/OPX.0b013e3182504346

[3] Reinstein DZ, Archer TJ, Gobbe M (2009) Corneal epithelial thickness profile in the diagnosis of keratoconus. Journal of refractive surgery 25: 604–610. 19662917 10.3928/1081597X-20090610-06

[4] Reinstein DZ, Srivannaboon S, Gobbe M (2009) Epithelial thickness profile changes induced by myopic LASIK as measured by Artemis very high-frequency digital ultrasound. Journal of refractive surgery 25: 444–450. 19507797 10.3928/1081597x-20090422-07 PMC2695568

[5] Rocha KM, Perez-Straziota CE, Stulting RD, Randleman JB (2013) SD-OCT analysis of regional epithelial thickness profiles in keratoconus, postoperative corneal ectasia, and normal eyes. J Refract Surg 29: 173–179. 23446013 10.3928/1081597X-20130129-08 PMC4123636

[6] Kanellopoulos AJ, Aslanides IM, Asimellis G (2012) Correlation between epithelial thickness in normal corneas, untreated ectatic corneas, and ectatic corneas previously treated with CXL; is overall epithelial thickness a very early ectasia prognostic factor? Clin Ophthalmol 6: 789–800. 22701079 10.2147/OPTH.S31524 PMC3373227

[7] Reinstein DZ, Gobbe M, Archer TJ, Silverman RH, Coleman DJ (2010) Epithelial, stromal, and total corneal thickness in keratoconus: three-dimensional display with artemis very-high frequency digital ultrasound. J Refract Surg 26: 259–271. 20415322 10.3928/1081597X-20100218-01 PMC3655809

[8] Reinstein DZ AT, Gobbe M (2008) Epithelial thickness in the normal cornea: three-dimensional display with Artemis very high-frequency digital ultrasound. Journal of refractive surgery 24: 571–581. 18581782 10.3928/1081597X-20080601-05 PMC2592549

[9] Reinstein DZ, Gobbe M, Archer TJ, Couch D (2011) Epithelial thickness profile as a method to evaluate the effectiveness of collagen cross-linking treatment after corneal ectasia. J Refract Surg 27: 356–363. 20954581 10.3928/1081597X-20100930-01

[10] Li HF, Petroll WM, Moller-Pedersen T (1997) Epithelial and corneal thickness measurements by in vivo confocal microscopy through focusing (CMTF). Current eye research 16: 214–221. 9088737 10.1076/ceyr.16.3.214.15412

[11] Sin S, Simpson TL (2006) The repeatability of corneal and corneal epithelial thickness measurements using optical coherence tomography. Optometry and vision science 83: 360–365. 16772894 10.1097/01.opx.0000221388.26031.23

[12] Prakash G, Agarwal A, Mazhari AI, Chari M, Kumar DA, et al (2012) Reliability and reproducibility of assessment of corneal epithelial thickness by fourier domain optical coherence tomography. Invest Ophthalmol Vis Sci 53: 2580–2585. 22427573 10.1167/iovs.11-8981

[13] Li Y, Tan O, Brass R, Weiss JL, Huang D (2012) Corneal epithelial thickness mapping by Fourier-domain optical coherence tomography in normal and keratoconic eyes. Ophthalmology 119: 2425–2433. 22917888 10.1016/j.ophtha.2012.06.023 PMC3514625

[14] Huang D, Swanson EA, Lin CP (1991) Optical coherence tomography. Science 254: 1178–1181. 1957169 10.1126/science.1957169 PMC4638169

[15] Eng J (2003) Sample Size Estimation: How Many Individuals Should Be Studied? 1. Radiology 227: 309–313. 12732691 10.1148/radiol.2272012051

[16] Haque S, Simpson T, Jones L (2006) Corneal and epithelial thickness in keratoconus: a comparison of ultrasonic pachymetry, Orbscan II, and optical coherence tomography. Journal of refractive surgery 22: 486–493. 16722488 10.3928/1081-597X-20060501-11

[17] Haque S, Jones L, Simpson T (2008) Thickness mapping of the cornea and epithelium using optical coherence tomography. Optometry and vision science 85: E963–976. 18832971 10.1097/OPX.0b013e318188892c

[18] Correa-Perez ME, Lopez-Miguel A, Miranda-Anta S, Iglesias-Cortinas D, Alio JL, et al (2012) Precision of high definition spectral-domain optical coherence tomography for measuring central corneal thickness. Invest Ophthalmol Vis Sci 53: 1752–1757. 22395881 10.1167/iovs.11-9033

[19] Reinstein DZ, Archer TJ, Gobbe M (2012) Refractive and topographic errors in topography-guided ablation produced by epithelial compensation predicted by 3D Artemis VHF digital ultrasound stromal and epithelial thickness mapping. J Refract Surg 28: 657–663. 22947295 10.3928/1081597X-20120815-02

[20] Schlegel Z, Hoang-Xuan T, Gatinel D (2008) Comparison of and correlation between anterior and posterior corneal elevation maps in normal eyes and keratoconus-suspect eyes. J Cataract Refract Surg 34: 789–795. 18471634 10.1016/j.jcrs.2007.12.036

[21] Chen X, Stojanovic A, Zhou W, Utheim TP, Stojanovic F, et al (2012) Transepithelial, Topography-guided Ablation in the Treatment of Visual Disturbances in LASIK Flap or Interface Complications. J Refract Surg 28: 120–126. 21974725 10.3928/1081597X-20110926-01

[22] Li Y, Meisler, D M, Tang M, Lu AT, Thakrar V, Reiser BJ, Huang D (2008) Keratoconus diagnosis with optical coherence tomography pachymetry mapping. Ophthalmology 115: 2159–2166. 18977536 10.1016/j.ophtha.2008.08.004 PMC2652571

[23] Du C, Wang J, Cui L, Shen M, Yuan Y (2012) Vertical and horizontal corneal epithelial thickness profiles determined by ultrahigh resolution optical coherence tomography. Cornea 31: 1036–1043. 22357393 10.1097/ICO.0b013e31823f8d56 PMC3366158

