# Supplementary material for: Comparison of Corneal Epithelial and Stromal Thickness Distributions between Eyes with Keratoconus and Healthy Eyes with Corneal Astigmatism ≥2.0 D
Source: PLoS One. 2014 Jan 28;9(1):e85994. doi: 10.1371/journal.pone.0085994 (PMC3904857; doi:10.1371/journal.pone.0085994)
Supplement: Protocol S1 — Trial protocol. (PDF) [file pone.0085994.s001.pdf]

Comparison of corneal epithelial and stromal thickness distribution between eyes with keratoconus and healthy eyes with corneal astigmatism >2.0 D

Principal Investigator, Research Team, and Study Site:

Principal investigator: Aleksandar Stojanovic

Co-Investigator: Wen Zhou

Contact Information: aleks@online.no; tel. +47 90693319

Study site: Øyeavdelingen UNN and SynsLaser Kirurgi AS, Tromsø

Lists of abbreviation

OCT: optical coherence tomography
SD: spectral domain
CXL: corneal collagen crosslinking

Study Population:

SD-OCT corneal scans of 20 eyes with keratoconus (group one) and 20 healthy eyes with > 2 diopters (D) of corneal astigmatism as controls (group two) will be studied. Keratoconus patients referred to the Eye Dpt. of the University Hospital North Norway for CXL-treatment and healthy subjects seeking preoperative evaluation for refractive surgery at SynsLaser Kirurgi AS Tromsø will be examined med corneal SD-OCT as a part of their routine examination. Analysis of their SD-OCT corneal scans will be used for the purpose of this study.

Study Design:

Cross-sectional, non-interventional, observational study

Sample Size:

Total number of participants: 40

Study Duration:

May 1 to October 31, 2013

Primary Objective:

To characterize epithelial and stromal thickness distribution in keratoconic eyes

Secondary Objectives:

To develop epithelial and stromal thickness–based variables for keratoconus detection

Background and Significance:

Corneal epithelium is a moldable[1] and active corneal layer that keeps the optical quality of the eye by remodeling itself to compensate for any change in the stromal surface shape e.g. after keratorefractive surgery[2, 3] or in keratoconus[2, 4-6]. Mapping of the corneal epithelial thickness may help detect stromal surface irregularities like subclinical cones, before they become detectable on topography[2, 4, 6].

Mapping of the corneal epithelium has been previously attempted by use of various technologies such as ultrasound, confocal microscopy[2, 4, 7] and Optical coherence tomography (OCT)[1, 3, 5, 8, 9].

OCT has been developed for non-contact, cross-sectional imaging in biological systems by using low-coherence interferometry to produce a two-dimensional image of optical scattering from internal tissue microstructures in a way that is analogous to ultrasonic pulse-echo imaging[10]. Spectral domain (SD), a newer generation OCT to be used in the current study, seems to be reliable and reproducible[3, 9] in measuring corneal epithelial thickness in contrast to the measurements with the time domain OCT[11] and does not require immersion like very-high-frequency digital-ultrasound, which is the only method providing sufficient resolution for corneal epithelial imaging[2, 4, 12-14].

A recently published study utilizing the SD-OCT technology[8] compares the epithelial thickness distribution of keratoconic corneas with the corneas of healthy subjects with unspecified amount of astigmatism, while the current study will use healthy corneas with > 2D of astigmatism as the control group.

Purpose of the study:

To characterize epithelial and stromal thickness distribution in keratoconic eyes and to develop epithelial and stromal thickness–based variables for keratoconus detection.

Methodology:

SD-OCT scans of 20 eyes with keratoconus (group one) and twenty healthy eyes with > 2 diopters (D) of corneal astigmatism as controls (group two) will be analyzed. Two cross-sectional meridional corneal profiles (along steepest and flattest meridians, located by a Scheimpflug-based corneal topography) for each individual will be obtained by using a RTVue100 (Optovue Inc. Fremont, California, USA), 26000-Hz SD-OCT across the central 6mm diameter of the cornea.

To map the epithelial and stromal thickness distribution in eyes from group one, 11 locations will be measured: corneal vertex, cone apex, mid-peripheral cornea (1.5mm from corneal vertex) and peripheral cornea (2.5mm from corneal vertex) along both the steepest and flattest meridian, and the locations with thickest epithelium along the steepest meridian. In eyes from group two, 11 corresponding locations will be measured.

Inclusion /Exclusion Criteria

SD-OCT scans of 20 confirmed cases of keratoconus referred to CXL treatment at the University Hospital North Norway and 20 healthy subjects seeking refractive surgery at SynsLaser Kirurgi AS in Tromsø for astigmatism >2 D will be used. The participants will be the subjects to the inclusion/exclusion criteria by the two institutions, regarding keratoconus and candidacy for refractive surgery, respectively.

Study Schedule:

SD-OCT scans obtained from May 1, 2013 through August 31, 2013 will be analyzed.

Statistical Analysis Plan:

Statistical analysis will be performed using SPSS 13.0 software. The independent-samples t test will be used to assess the difference of epithelial and stromal thickness at corresponding locations between two groups and between different locations within group.

Descriptive statistics will be carried out for all eyes using vertical mirrored symmetry superimposition: thickness values for left eyes will be reflected in the vertical axis and superimposed onto the right eye values so that nasal/temporal characteristics could be combined.

The sample size was decided by the power calculation founded on data from the previous studies analyzing corneal epithelial thickness in keratoconus and healthy eyes. For a significance level of 0.05 and a statistical power of 0.8, the equation for sample size is: $N=31.4x\sigma^2/D^2$, where σ is assumed standard distribution of each group and D is the minimum expected difference between two means. According to the preliminary data collected from comparable studies[4], population σ and D are chosen to be 5.0 and 4.7 respectively, yielding a sample size N=35.5. In the current study we chose a sample size 40.

Informed Consent Process:

The research will comply with the tenets of the Declaration of Helsinki. Informed consent will be obtained ahead of the examination. Invitation to participate in the scientific study and informed consent (both written in Norwegian) will be sent to prospective study participants in the letter of notifications for the scheduled visits to the Eye Dpt. UNN and to the SynsLaser Kirurgi AS in Tromsø, respectively.

Privacy and confidentiality: Participant's names will be kept on a password-protected database, and will be linked only with a study identification number for this research. All the research data will be entered into a computer that is password protected and will be stored in a locked office of the investigators.

Risk/Benefit
Risk to participants

The participants in the project will be recruited either among the patients with keratoconus referred to the Eye Department, UNN for general or pre-CXL evaluation,

or among the candidates seeking refractive surgery at the SysnLaser Kirurgi AS in Tromsø. The current study will not interfere with, or add any extra examinations to the participants´ already planned diagnostic/treatment procedures, thus it will not bring any extra risks to the participants.

Benefits to participants

The study will lead to better pre-corneal refractive surgery candidacy screening and potentially better postoperative result, and it may lead to earlier diagnosis of keratoconus.

Conflict of interest

None of the investigators involved in the current study has financial interest with the products mentioned in the study.

Publication and presentation plans

The results of the study will be subject to presentations at various national and international congresses and provide the basis for manuscripts submission to peer reviewed journals of ophthalmology and refractive surgery.

Data will be submitted for publication between September and October 2013.

References:

1. Lian Y, Shen M, Jiang J, et al. Vertical and horizontal thickness profiles of the corneal epithelium and Bowman's layer after orthokeratology. Investigative ophthalmology & visual science 2013;54(1):691-6.
2. Reinstein DZ, Srivannaboon S, Gobbe M, et al. Epithelial thickness profile changes induced by myopic LASIK as measured by Artemis very high-frequency digital ultrasound. Journal of refractive surgery 2009;25(5):444-50.
3. Schmoll T, Unterhuber A, Kolbitsch C, et al. Precise thickness measurements of Bowman's layer, epithelium, and tear film. Optometry and vision science : official publication of the American Academy of Optometry 2012;89(5):E795-802.
4. Reinstein DZ, Archer TJ, Gobbe M. Corneal epithelial thickness profile in the diagnosis of keratoconus. Journal of refractive surgery 2009;25(7):604-10.
5. Rocha KM, Perez-Straziota E, Stulting RD, Randleman JB. SD-OCT analysis of regional epithelial thickness profiles in keratoconus, postoperative corneal ectasia, and normal eyes. Journal of refractive surgery 2013;29(3):173-9.
6. Kanellopoulos AJ, Aslanides IM, Asimellis G. Correlation between epithelial thickness in normal corneas, untreated ectatic corneas, and ectatic corneas previously treated with CXL; is overall epithelial thickness a very early ectasia prognostic factor? Clinical ophthalmology 2012;6:789-800.
7. Li HF, Petroll WM, Moller-Pedersen T, et al. Epithelial and corneal thickness measurements by in vivo confocal microscopy through focusing (CMTF). Current eye research 1997;16(3):214-21.

8. Li Y, Tan O, Brass R, et al. Corneal epithelial thickness mapping by Fourier-domain optical coherence tomography in normal and keratoconic eyes. Ophthalmology 2012;119(12):2425-33.
9. Prakash G, Agarwal A, Mazhari AI, et al. Reliability and reproducibility of assessment of corneal epithelial thickness by fourier domain optical coherence tomography. Investigative ophthalmology & visual science 2012;53(6):2580-5.
10. Huang D, Swanson EA, Lin CP, et al. Optical coherence tomography. Science 1991;254(5035):1178-81.
11. Sin S, Simpson TL. The repeatability of corneal and corneal epithelial thickness measurements using optical coherence tomography. Optometry and vision science : official publication of the American Academy of Optometry 2006;83(6):360-5.
12. Reinstein DZ, Archer TJ, Gobbe M. Refractive and topographic errors in topography-guided ablation produced by epithelial compensation predicted by 3D Artemis VHF digital ultrasound stromal and epithelial thickness mapping. Journal of refractive surgery 2012;28(9):657-63.
13. Reinstein DZ, Archer TJ, Gobbe M, et al. Epithelial thickness in the normal cornea: three-dimensional display with Artemis very high-frequency digital ultrasound. Journal of refractive surgery 2008;24(6):571-81.
14. Reinstein DZ, Gobbe M, Archer TJ, Couch D. Epithelial thickness profile as a method to evaluate the effectiveness of collagen cross-linking treatment after corneal ectasia. Journal of refractive surgery 2011;27(5):356-63.
